# Decolonising against a backdrop of colonial amnesia: barriers, challenges, and finding a way forward

**DOI:** 10.3389/fsoc.2025.1554679

**Published:** 2026-01-23

**Authors:** Javeria Khadija Shah

**Affiliations:** Royal Central School of Speech and Drama, University of London, London, United Kingdom

**Keywords:** decolonisation, colonial amnesia, whiteness ecology theory, eurocentrism, self-decolonisation, autoethnography, structural transformation

## Abstract

This paper, originally delivered as a keynote at De Montfort University, interrogates the persistence of colonial amnesia within educational, institutional, and cultural contexts in the UK. Through an autoethnographic lens, it explores both structural and embodied barriers to meaningful decolonisation, drawing attention to the epistemic violence of historical erasure alongside the deeply personal labour of self-decolonisation. Combining conceptual critique with situated narrative, the paper presents three autoethnographic vignettes that examine naming, diasporic dissonance, and joy as a mode of refusal. It argues for a dual praxis that foregrounds structural transformation while simultaneously centring introspective reclamation. The analysis ultimately underscores the need for healing, justice, and historical redress within ongoing struggles for equity and recognition.

## Introduction and context

This paper extends from a keynote I delivered at De Montfort University in 2024, during a period marked by intensifying revisionist histories, state-sanctioned cultural amnesia, and the quiet retreat of institutions from their earlier commitments to racial justice. The speech, and this paper, emerged as both a provocation and a reflection. How do we make sense of the persistent forgetting of colonial violence within education, policy, and public memory in Britain? Moreover, how do those of us from historically colonised communities navigate this forgetting while carrying its scars?

My focus is colonial amnesia: a systemic form of erasure in which the atrocities and logics of empire are selectively sanitised, softened, or omitted from dominant discourse. However, this forgetting is not passive. It is active, curated, and performative. Drawing on scholars such as Stahn (2021), Tuck and Yang (2012), and Smith (2012), this paper contends that colonial amnesia not only distorts historical truth but also impedes future justice by severing the link between violence and restitution.

As a British-born academic of Panjabi Pakistani heritage who is visibly Muslim, spiritually rooted, and professionally embedded within UK higher education, I do not approach this topic from neutrality. I write from the intersections of race, gender, class, and religious visibility. My body, name, and narrative are not abstract. They are situated within the very institutions that enact, obscure, and at times perform progress. This work is therefore both personal and political.

To ground this analysis, I draw on autoethnography as an epistemic and decolonial method. I offer three vignettes: reclaiming my name, confronting diasporic dissonance in Pakistan, and framing joy as a form of resistance. These are not asides. They are a method. They illuminate how coloniality operates not only through systems and institutions but also through syllables, silences, and stolen softness.

This paper argues that decolonisation must move beyond curriculum change or symbolic gestures. It must involve confronting structural whiteness, excavating silenced histories, and restoring epistemic and emotional sovereignty to those who have long been dispossessed. This is not a linear project. It is iterative, embodied, and necessarily uncomfortable. As Gaztambide-Fernández (2012) asserts, decolonisation is a collective pedagogical effort built on relationality, solidarity, and refusal. This aligns with the pursuit of self-decolonisation through autoethnography, as well as educational reform initiatives that directly challenge colonial epistemologies (Andreotti, 2011).

In navigating this terrain, I also introduce my theoretical contribution: a framework I have termed the ecology of whiteness (Shah, 2021). This theory interrogates whiteness not as an identity but as an environment. This permeable, regulating force shapes how melanated bodies are read, regulated, and resisted across institutional and cultural life.

The structure of this paper is as follows. Section II outlines the methodological grounding in autoethnography as decolonial praxis. Sections III through V present three vignettes that ground the theory in lived reality. Section VI addresses the broader structural and ideological mechanisms of colonial amnesia, while Section VII explores practical and theoretical pathways forward. The paper concludes with a call to action—not merely to decolonise texts or policies, but to undertake the deeper, riskier work of re-membering and reassembling that which has been intentionally fragmented.

### A note on form: writing as decolonial refusal

The structure and voice of this paper are themselves a deliberate act of decolonial resistance. Rather than conforming to the sanitised, depersonalised style of dominant academic discourse, I embrace an autoethnographic voice rooted in vulnerability, ancestral memory, and the political power of storytelling. As scholars such as Smith (2012), hooks (1994), and Thiong’o (1986) remind us, reclaiming voice, rhythm, and personal narrative within scholarly writing is a form of resistance to epistemic violence. It challenges the Eurocentric norms that dismiss lived experience as anecdotal, and it centres the embodied knowledge of those who have been historically written about but rarely allowed to write. This paper, therefore, not only discusses decolonisation. It attempts to enact it through form, cadence, and content. The inclusion of vignettes, spiritual reflections, and memory fragments is intentional. They are not aside; they are the methodology.

### Methodological framing: autoethnography and the ecology of whiteness

This paper adopts autoethnography not as literary flourish, but as a critical epistemological intervention (Adams et al., 2015). Within the context of decolonial scholarship, autoethnography disrupts the illusion of objectivity that often undergirds Eurocentric research paradigms. It treats the self not as an isolated reference point, but as a socially and historically situated locus through which larger institutional, cultural, and political structures are lived, felt, and confronted. As Tuhiwai Smith (2012) reminds us, methodologies are never neutral. They are underpinned by ontologies and epistemologies that reflect particular worldviews. In this light, autoethnography becomes both a method and a message—an embodied refusal of the methodological erasures that have long dismissed colonised subjects as unknowing, unspeaking, or irrelevant.

To question autoethnography’s legitimacy is to expose the knowledge hierarchies it threatens. Its critics frequently draw on a language of rigour and neutrality, invoking terms shaped by masculinist, colonial, and positivist traditions that discredit the personal as somehow unacademic. Yet, as Boylorn and Orbe (2014) and Augustus (2022) insist, it is precisely this centring of lived experience, affective truth, and relational accountability that renders autoethnography both radical and rigorous. For scholars and practitioners writing from racialised, gendered, and marginalised locations, it offers not just methodology, but survival. It becomes a way of re-entering the narrative on one’s own terms, reclaiming space in archives and institutions that have long sought to define us in absentia.

In approaching this work, I build also on Chakrabarty’s (2000) critique of historicism and Loomba’s (2005) articulation of coloniality and its aftermath. Both scholars call us to question the temporal and spatial norms through which knowledge is produced and legitimised. Who defines historical relevance? Who determines epistemic legitimacy? When Spivak (1988) posed her pivotal question, “Can the subaltern speak?,” she was not asking simply whether voice is possible, but whether it is ever permitted to exist outside frameworks of translation. In contemporary research cultures, this question remains pressing. Too often, racialised knowledge must be reformulated in the grammar of the dominant in order to be heard, let alone valued.

The present study therefore engages autoethnography as both a decolonial method and a site of theoretical inquiry. I offer three vignettes—on naming, diasporic dissonance, and joy as refusal—not as tangential reflections, but as key sites of analysis. These moments are data. They illuminate the intimate mechanisms through which coloniality is enacted and endured. A mispronounced name in a seminar, a lingering colonial accent in a school in Lahore, the refusal to forfeit softness in a system that rewards only performance—these are not anecdotes. They are analytical entry points into understanding how colonial amnesia operates not only across curricula and institutions, but through bodies, silences, and social atmospheres.

This methodological grounding is also the context in which I introduce my conceptual framework: the ecology of whiteness (Shah, 2021). Drawing from years of navigating white-majority educational and cultural spaces, this theory treats whiteness not solely as ideology or identity, but as an ambient structure—a regulating environment that shapes perception, belonging, and affect. In *An Ecological Exploration of Whiteness*, I frame whiteness as something atmospheric. It surrounds and conditions. It is enacted not only through policy, but through silence, comfort, and the unspoken architectures of legitimacy. It disciplines space, time, emotion, and the very notion of what constitutes the ‘proper’ academic subject.

The ecology of whiteness provides a way to think about whiteness as terrain—at once spatial, psychic, procedural, and affective. Within this terrain, those of us from Global Majority backgrounds are continuously asked to calibrate our presence. Hypervisibility, tokenism, unspoken dress codes, veiled resistance to difference: all become part of the atmospheric negotiation. Autoethnography enables these dynamics to surface. It renders what is ambient visible. It names what is often considered ineffable. In doing so, it refuses the aesthetic of detachment that continues to define much of academic writing.

In contexts where colonial amnesia functions not only through curriculum design but through national silence and cultural disavowal, autoethnography provides a means to write oneself back into the record. To acknowledge what has been erased is not a personal indulgence; it is a scholarly and political act. This work asserts that the knowledge of the historically colonised need not pass through the filter of the dominant in order to be credible.

In writing this paper, I offer more than an analytic frame. I offer testimony. The vignettes that follow are partial, situated, and specific. They are not presented as representative. Rather, they are positioned to make visible the epistemic, affective, and institutional patterns that colonial forgetting relies upon. By inscribing these fragments into academic space, I participate in a wider project of reconstitution. This is not only a method. It is a way of remembering differently.

## Autoethnographic vignettes: memory, erasure, and embodied resistance

### Vignette I: reclaiming the name

There was a time when I introduced myself as “Jo.” It was a survival tactic—an anglicised shield forged in classrooms, interview panels, and institutional corridors where difference was noticed but never comfortably named. “Jo” was more manageable, softer, and most importantly, safer. However, safety is not the same as sovereignty.

It took decades to reclaim my full name: Javeria Khadija Shah. Each syllable is sacred. “Javeria,” drawn from the name of a wife of the Prophet Muhammad ﷺ, connects me to faith, strength, and prophetic womanhood. “Khadija” invokes Sayyidah Khadija al-Kubra (r.a), the first to believe, the first to build. Moreover, “Shah,” a Persian title of kingship and spiritual nobility, carries with it both the burden and the beauty of ancestral legacy. Though common in South Asia today, its origins are deeply Iranian, echoing Sufi lineages and Indo-Persian cultural memory. I did not choose this name; it chose me. However, reclaiming it was a choice. A decolonial act. A spiritual return.

What followed that act, however, has been quietly instructive. Since returning to my given name in full, on email signatures, author bylines, and conference bios, I have noticed a discernible decline in mainstream invitations, collaborations, and institutional opportunities. The shift was not announced, but it was felt. Rooms that once welcomed me under my anglicised name grew quieter. The gatekeeping became subtler. My presence, now fully present, seemed to unsettle the aesthetic norms of inclusion.

Even within progressive spaces, the name “Javeria Khadija Shah” is often truncated, mispronounced, or replaced by an acronym. These are not minor slips. They are micro-performances of erasure. As Ahmed (2012) might argue, such repetitions reinforce a conditional form of belonging, where visibility is permitted only when it does not disturb the comfort of the dominant group.

Naming, for me, is more than semantics. It is methodology. It is resistance. Through the *My Name Is* project, I have invited others to explore the politics of their names—what was lost, what was altered, what was proudly reclaimed. This work revealed a pattern: names from melanated and Muslim cultures are often corrected, compressed, or softened to soothe the white gaze. However, they are also sites of memory, of inheritance, of refusal.

To reclaim one’s name is to return to oneself, not the self-imagined by empire, but the one whispered by ancestors, written in duʿāʾ, and remembered in full. It is to accept that such a return may come at a cost, and to walk the path anyway.

### Vignette II: naming the self in a world that erases

This vignette draws on autoethnographic methodology, engaging religious practice as a site of epistemic resistance and cultural memory (Smith, 2012; Mahmood, 2005; Boylorn and Orbe, 2014), and includes terms such as “Sayyida” to signal reverence within Islamic tradition. There is something profoundly political about a name. In the aftermath of empire, names are never incidental. They are signifiers of inheritance and markers of permission. They determine who is seen, how we are remembered, and which parts of us must be softened to be heard. For years, I navigated academia and the creative industries using a version of my name that softened my differences. It made my presence more palatable to white audiences, facilitated easier introductions, and eliminated what one colleague once referred to—without irony—as “linguistic friction.” However, in recent years, I made a deliberate decision to return fully to my original name: Javeria Khadija Shah. This was not a rebranding. It was a homecoming. My name is not a performance. It is not a symbol of resistance simply for the sake of provocation. It is a declaration of return to ancestral dignity, to matrilineal power, to the rich Indo-Persian spiritual and cultural legacy from which I come. It is the name of Sayyida and storytellers, a name inscribed with *barakah* (*a concept in Islamic tradition referring to divine blessing and sacred continuity*). A name too often misread.

Even within institutions where I am explicitly known for my work on coloniality and naming politics, there is palpable resistance. Emails are addressed to “Dear Javeria Shah,” omitting Khadija, even when the full name is provided. Event flyers shorten or modify my name without my consent. Citations are published with errors. These are not administrative oversights; they are semiotic strategies of containment.

Through the *My Name Is* project, I have witnessed the widespread and intimate nature of these patterns. The names of racialised women—especially those of Muslim and Global Majority heritage—are trimmed, anglicised, or joked about, often as part of what passes for “banter.” This erasure becomes normalised, expected. However, in reclaiming our names, we disrupt the rhythm. We shift the gaze. We refuse the small violence.

To name oneself, entirely and without apology, is a revolutionary act in a world that still prefers our edits. My name, now spoken whole, carries not just personal truth but spiritual continuity. In refusing to be renamed, I reclaim the right to tell my own story.

### Vignette III: diasporic dissonance in the shadow of empire

Diaspora is often romanticised as a bridge between two worlds, but for many of us, it is a wound held open by history. Travelling to Pakistan, I am frequently struck by how empire lingers in the bones of the landscape. The signage in affluent neighbourhoods is in English. The architecture of state buildings is Victorian. Elite schools market Canadian or British curricula as the gold standard. Even success is imagined as a flight path toward London, Toronto, and Sydney. Language becomes a crucial terrain of struggle. As Thiong’o (1986) asserts, the colonisation of the mind is maintained through linguistic dominance; diasporic subjects often internalise English as a measure of intelligence or civility while suppressing their own mother tongues and cultural references. This terrain is historically structured through Eurocentric knowledge systems that frame linguistic assimilation as progress and mark Global South vernaculars as inferior (Blaut, 1993; Bhambra, 2014). This is further echoed in Said’s (1978) critique of Orientalism and Young’s (2001) historical tracing of how colonial discourse constructed non Western languages and knowledge systems as peripheral.

As a British-born Pakistani, I feel this dissonance intensely. The land that should feel like home is shaped by the same colonial logics I resist in the UK. Whiteness as an aspiration remains embedded in institutional norms, media aesthetics, and economic mobility. The English language—once the tongue of the coloniser—still defines prestige. This is what I have come to call diasporic dissonance: the ache of recognising that the epistemologies you seek to escape are replicated across borders.

Partition is remembered politically in Pakistan, but coloniality as a knowledge system is rarely interrogated. This dissonance leaves little space for ancestral wisdom to breathe. It becomes challenging to reimagine futures when the present remains tethered to imperial templates.

To decolonise, then, is not just to critique the West—it is to resist the *internalisation* of its gaze. It is worth remembering that spiritual grandeur, artistic brilliance, and intellectual sovereignty once flourished on our soil, long before borders and bureaucracies redefined what was valuable. The work of reclamation must therefore happen on both sides of the sea.

### Vignette IV: before borders: ancestral memory, colonial fracture, and the refusal of erasure

My family were not muhājirīn (Arabic: migrants; often used to describe post-Partition Muslim families who moved to Pakistan) in the post-Partition sense. We did not *migrate* in the way history often flattens us. Instead, the lands around us were renamed, redrawn, and divided—turning neighbours into foreigners and heritage into a category. We were already there. We belonged to the land before the maps changed.

Our ancestral threads stretch from the sacred cities of Makkah and Madinah, through Iraq and Khazwain—an ancient region near the Caucasus—down to India, where spiritual trade routes and sacred lineages rooted us deeply in the soil. As colonial rule intensified, the British Raj fractured our family, dispersing us not by choice, but by structure. Some were pushed toward Kenya, where they became caretakers of civic institutions; others remained in India or were caught in the aftermath of Partition as it unfolded into Pakistan. Some were eventually rerouted to Britain.

This was not a single act of movement, but a series of ruptures—an empire’s aftershock recorded in the lives of brown families scattered across three continents.

However, we were not migrants in the way the Empire named us. We were witnesses—bearers of sacred continuity. Our movement carried prayer, language, memory, and craft, not simply survival. What we inherited was not loss, but barakah scattered like stars across colonised skies.

In reclaiming this story, I reject the frame of arrival. We did not *arrive* in Britain—we were processed, sorted, and subdued. However, we were always *present*. Our names were written long before yours mispronounced them.

### Vignette V: reclaiming the veil—a decolonial Reading of the hijab

The decision to begin wearing the hijab full-time 2 years ago was not sudden, nor was it solely spiritual; it was, and remains, a complex act of epistemic resistance. In the context of colonial modernity, veiling has often been misread through the lens of oppression, backwardness, or excess religiosity. Historically, the practice of head covering has been present across various traditions, including Catholic nuns, Orthodox Jewish women, and even depictions of the Virgin Mary, which have all adopted veiling as a symbol of piety and dignity. The colonial erasure of these parallels has contributed to the exceptionalisation and vilification of Muslim women’s veiling.

My hijab journey is shaped by both ancestral memory and contemporary politics. As a visibly Muslim woman of Pakistani heritage, the act of covering became not just about faith, but about confronting a system that demands assimilation while fearing difference. In classrooms, boardrooms, and academic panels, my visibility as a hijab-wearing academic often elicits discomfort, curiosity, or polite silence. These micro-responses reveal the persistent Eurocentric lens through which Muslim women are read, either as threats to liberal values or as symbols in need of saving.

However, the veil, for me, is neither submission nor spectacle. It is a sacred boundary, a politicised aesthetic, and a decolonial stance. It is a commitment to self-definition on my terms, one that refuses the colonial gaze and recentres embodied agency. I do not wear it to make a statement, but it inevitably becomes one, especially in institutions that claim to be inclusive while enforcing uniformity. “I cover my head not to disappear, but to be fully seen without distortion, without translation.” In this sense, the hijab is part of a broader decolonial project: reclaiming narrative, resisting erasure, and making visible the multiplicity of ways to be, to believe, and to belong.

### Vignette VI: joy as resistance

Amidst the exhaustion of navigating erasure, it would be easy to become hardened. However, I have learned—sometimes painfully, sometimes miraculously—that joy is a decolonial inheritance. It is not the absence of struggle. It is the refusal to be reduced by it.

There is joy in saying my name aloud, entirely. In wearing hijab, it is not a matter of concealment, but rather a covenant. In laughing without self-surveillance. In writing my truths with softness and strength. These are acts of survival, yes, but more than that, they are acts of refusal. My joy, I have come to understand, is an act of resistance. It is a refusal to be diminished. As hooks (1981) wrote in Ain’t I a Woman?, the survival of Black and Brown women in the face of structural oppression is not merely endurance—it is testimony. This radical joy sits at the heart of my decolonial praxis.

I refuse to let colonial infrastructures of forgetting define how I see myself. I refuse to let white-coded professionalism steal my cadence, my warmth, my rhythm. I refuse to let institutional fragility eclipse my sacred clarity. I will not surrender my tenderness to their systems. I will not be deterred by being digestible.

In a world that demands self-regulation from Global Majority women, feeling joy is a reclaiming of what was taken. My joy is prayer. It is an inheritance. It is medicine. Moreover, it is mine.

### Naming colonial amnesia

Colonial amnesia is not merely the absence of memory. It is a structured and intentional form of forgetting, designed to obscure the violences of empire while protecting the comfort and convenience of dominant groups. This forgetting is not passive. It is curated and institutionalised, operating discursively and materially across curricula, media, governance, and collective consciousness. Through this architecture, colonialism is repackaged as benevolence, resistance is erased from memory, and the need for reparation is quietly neutralised (Shilliam, 2017; Acheraiou, 2020).

Following Stahn’s (2021) conceptualisation, I understand colonial amnesia as a systemic epistemic distortion (Acheraiou, 2020). It functions to suppress truths that are politically inconvenient, particularly those which challenge the myth of imperial virtue. The effect is not only historical. This curated silence distorts current understandings of justice and accountability, severing the links between historical violence and contemporary inequality.

Education is a key terrain where this amnesia becomes visible. In England, the national curriculum continues to frame empire through narratives of industrial innovation, wartime sacrifice, and national pride. There is minimal engagement with the extractive logics and racialised violence (Anderson, 1983) underpinning Britain’s imperial legacy (Andrews, 2018). As a result, students from Global Majority backgrounds often find their histories excluded or distorted. They must either seek them elsewhere or internalise the message that their communities have no history worth knowing.

The media, too, plays a central role in maintaining this amnesia. Racialised trauma is either hyper-visible in moments of spectacle or rendered invisible in the routine operations of policy and discourse. Public debates on immigration, national identity, or social cohesion rarely acknowledge the entanglement of Britain’s domestic landscape with its colonial past (Gilroy, 1993; Fletcher, 2012). Empire has not disappeared. It has simply been renamed (Baker and Cupery, 2023).

What sustains this forgetting is not only institutional silence, but an affective economy that rewards patriotism and punishes critical reflection (Macdonald, 2009; El-Enany, 2020). Speaking openly about empire’s violence is labelled as divisive. Naming whiteness is described as aggressive. Demanding historical truth is framed as unpatriotic. This is not a neutral lapse in memory (Fraiture, 2022). It is a deliberate and political strategy designed to shield dominant narratives from scrutiny.

As Fanon (1963) warned, forgetting in the colonial context is itself a form of violence (Césaire, 2001). It enables continued psychic and material domination by denying the legitimacy of the oppressed and rewriting the terms of remembrance. Naming this forgetting is therefore not an act of nostalgia. It is a pursuit of justice.

### Whiteness and epistemic domination

White supremacy, historically legitimised through pseudo-science, theology, and geopolitical expansion, remains a foundational architecture of global knowledge systems. It is not confined to ideology alone. It functions as a cognitive ecology—a system of perception (Blaut, 1993; Moreton-Robinson, 2013), value, and affect that normalises whiteness as the universal standard while constructing all else as deviant, excessive, or unintelligible (Moreton-Robinson, 2015; Mills, 1997).

In my 2021 chapter *An Ecological Exploration of Whiteness*, I proposed that whiteness be understood not only as a cultural hegemony, but as an immersive social atmosphere. This ecology is sustained through racial socialisation, institutional conditioning, and imperial residues that shape individual behaviour and collective assumptions (Shah, 2021). Whiteness, viewed in this way, is absorptive and ambient. It conditions academic, cultural, and emotional climates, influencing hiring decisions, citation practices, curricular hierarchies, and everyday interactional norms. It adapts in response to critique, often cloaking itself in the language of diversity and inclusion without relinquishing structural power (Taylor, 2020; Saranillio, 2017).

Eurocentrism, within this ecology, operates as a cartographic principle (Bhambra, 2014). It presents the European experience as normative and Western epistemologies as universal. Knowledge systems from the Global South are then rendered either obsolete or reclassified as anecdotal, spiritual, or folkloric—acceptable only when translated into Western academic lexicons (Quijano, 2000).

This ideological order is reinforced along multiple axes (Said, 1978). Religious traditions are racialised, often dismissed or exoticised through colonial lenses. Economic systems reproduce racial hierarchies in global labour markets. Spatial hierarchies persist through the maintenance of colonial borders and development paradigms. Gender and sexuality are also configured by this ecology, as Lugones (2007) argued, through colonial modernity’s imposition of binary, heteropatriarchal norms (McClintock, 1995).

The ecology of whiteness is not fixed, but highly adaptive. It maintains itself through a combination of forgetting and reward. Those who forget the origins of their privilege are granted ease. Those who question the system encounter resistance, surveillance, or expulsion.

To theorise whiteness ecologically is to expose its sustainability. It requires asking difficult questions. What are the roots, nutrients, and toxins that allow it to persist, especially within institutions that profess inclusion? Who breathes easily in these spaces, and who is suffocated by their atmospheres? (Young, 2001).

Only by naming the environment that sustains whiteness can we begin the deeper work of transformation.

### Pathways for addressing colonial amnesia

Confronting colonial amnesia is not merely an exercise in historical inclusion. It demands a fundamental reckoning across structural, epistemic, and emotional registers. The following pathways are offered as entangled strategies that foreground both systemic transformation and the personal labour of decolonial return.

#### Self-decolonisation as praxis

The work of decolonisation must begin with the self. This does not mean adopting an individualist stance, but rather recognising that colonial violence is lived in the body, the memory, and the voice. Self-decolonisation requires the active unlearning of internalised inferiority. It calls us to reclaim our names, languages, epistemologies, and emotional registers. These acts are not symbolic. As the vignettes in this paper illustrate, they carry costs—economic, relational, and professional. However, they also anchor us in truths that institutions often refuse to name.

#### Embodied and marginalised knowledge at the centre

Decolonisation must expand the boundaries of what counts as legitimate knowledge. This includes honouring oral tradition, intergenerational memory, artistic and spiritual insight, and lived experience as valid forms of scholarship. It also involves transforming the institutional structures that gatekeep knowledge—particularly who teaches, what they teach, and how they are resourced. Decolonial pedagogy is not a matter of content alone. It is a question of positionality, methodology, and power.

#### Affective empathy and radical relationality

Colonial amnesia is not only intellectual. It is emotional. It dulls institutional empathy toward Global Majority communities and normalises detachment from harm. A decolonial future must be relational. This means cultivating educational, policy, and leadership spaces that prioritise care, humility, and collective responsibility. Efficiency alone cannot lead us to justice. Compassion, accountability, and memory must guide the way.

True decolonial work cannot be technocratic. It is spiritual, relational, and embodied. Without this depth, our interventions risk becoming performative rather than transformative.

## Conclusion: a return to self as praxis

To decolonise within the context of colonial amnesia is to work within silences that were never accidental. These silences were manufactured. This paper has argued that the labour of remembrance and reclamation must begin within ourselves—not as isolated individuals, but as carriers of ancestral knowledge, cultural dignity, and epistemic sovereignty.

My trajectory, as a British-born Pakistani Muslim woman, illustrates both the toll and necessity of this work. To reclaim one’s name, one’s dress, one’s voice, and one’s softness is not always welcomed in spaces shaped by whiteness. Yet the refusal to be renamed, re-coded, or subdued is a form of resistance that no institution can fully contain.

We must reimagine our classrooms, unearth silenced histories, and confront whiteness not only as a cultural ideology but as an ecological system that governs breath, space, and value. However, perhaps more urgently, we must affirm that joy, softness, and memory are not ornamental—they are strategies of survival. They are revolutionary technologies.

To remember, to name, to feel joyfully—these are not merely personal acts. They are, and have always been, political.
